# Congenital hypothyroidism after assisted reproductive technology in Japan: comparison between multiples and singletons, 2005–2009

**DOI:** 10.1186/1687-9856-2013-5

**Published:** 2013-02-12

**Authors:** Syuichi Ooki

**Affiliations:** 1 Department of Health Science, Ishikawa Prefectural Nursing University, 1-1, Gakuendai, Kahoku, Ishikawa, 929-1210, Japan

**Keywords:** Congenital hypothyroidism, Assisted reproductive technology, Multiple births, Live births, Epidemiologic study, Incidence rate, Concordance rate, Recurrence risk ratio, Familial aggregation

## Abstract

**Background:**

The relationship between congenital hypothyroidism (CH) and multiple pregnancy has not been fully studied in Japan.

**Methods:**

Complete nationwide data of assisted reproductive technology (ART) in Japan from 2005 to 2009 presented by the Japan Society of Obstetrics and Gynecology, which include information on CH and birth defects were used for analyses. Diseases were reclassified according to the International Classification of Diseases, tenth edition (ICD-10, 2003 version). The relative risk (RR) and 95% confidence interval (CI) of the incidence rate for CH was calculated with singletons as the reference group. Additional birth defects with CH were examined. The probandwise concordance rate of multiples and recurrence risk ratio were calculated.

**Results:**

There were 18 patients with CH, consisting of 12 singletons and 6 multiples. The incidence rates of CH per 1,000 live births in singletons and multiples are 0.14 (=12/87,145) and 0.31 (=6/19,533), respectively. The incidence rate was more than twofold higher in multiple births than in singleton births, but the difference was not statistically significant (RR=2.2, 95% CI 0.8–5.9). Additional birth defects were present in three patients with CH (17%=3/18). The multiples were affected by other birth defects more frequently (33%=2/6) than the singletons (8%=1/12). The six multiple-births patients were derived from one concordant twin pair, which consisted of two twin patients; three discordant twin pairs, which consisted of three twin patients; and one discordant triplets set, which consisted of one triplet patient. Thus, the probandwise concordance rate was 33.3% (=2/(2+4)). The estimated recurrence risk ratio was 1976 (for the total ART population) or 609 (for the Japanese general population).

**Conclusions:**

CH was more frequent in multiples compared to singletons. A higher percentage of additional birth defects was also observed in multiples compared to singletons. The familial aggregation of CH was suggested.

## Background

Congenital hypothyroidism (CH) is defined as thyroid hormone deficiency present at birth and is classified into permanent and transient CH [1]. An exact cause for the vast majority of cases of CH (or thyroid dysgenesis) remains unknown [1]. CH is a relatively common congenital disorder, occurring in about 1 of 1,500 to 4,000 live births [2-4]. The incidence rate of CH has been reported to be increasing [1,5,6]. The reasons for the increased incidence rate are not clear, but several possibilities have been pointed out, including a change in the screening test cutoff point [2], enhanced detection [5], the increase of preterm infants [1], and the misclassification of some cases of transient CH as permanent CH [7].

Several risk factors of CH have been presented in epidemiologic studies. Females are consistently reported to more frequently suffer from CH than males [1,8]. The association between birth weight and CH was reported to show a U-shaped curve [9]. According to a population-based case–control study in Italy by Medda et al. [10], the statistically significant risk factors for permanent CH were twins, additional birth defects, female gender, and gestational age >40 weeks. Using the same CH registry, Olivieri et al. [11] showed that the incidence rate of CH in multiples was more than 3-fold higher compared to singletons. Another population-based study in the U.S. also showed that the incidence rates of CH in twins doubled as compared to that in singletons, and the incidence rate was even higher with triplets/+ [6]. Moreover, many studies reported a higher incidence rate of extrathyroidal additional birth defects among neonates with CH compared with the general population [8,12-24], especially congenital heart disease [8,14,17,21-25].

Many case studies showed that CH occurs sporadically [26], although dyshormonogenic cases are often recessively inherited, and recent cohort analyses estimated that approximately 2% of cases with thyroid dysgenesis are familial [27,28]. Olivieri et al., in the only population-based twin study published to date [11], noted that the pairwise concordance rate of twin pairs for permanent CH was low (4.3%).

Given these circumstances, assisted reproductive technology (ART) data present a unique opportunity for twin study [29]. The percentage of multiple births among the ART population is much higher than that in the general population. Most twins after ART are dizygotic (DZ) siblings that develop together in the same womb. Thus, the aim of the present study was to compare the incidence rate of CH in multiples with singletons, examine additional birth defects with CH, and analyze the familial aggregation of CH using nationwide data on ART in Japan.

## Materials and methods

### Outline of Japanese birth defects data after ART

The method for collecting data is described in detail elsewhere [29]. Almost all medical institutions performing ART, which does not include simple ovulation stimulation/enhancement, are registered with the Japan Society of Obstetrics and Gynecology (JSOG). The JSOG administers questionnaire surveys for these medical institutions. Some of the survey data are presented in simple annual reports of aggregate, not individual, data. The individual list of all ART pregnancies resulting in congenital diseases, not all ART pregnancies, is presented every year in the JSOG annual reports on ART (in Japanese). The presented items are method of treatment, blastocyst transfer, maternal age, perinatal outcome and the gestational week, plurality, sex, early neonatal infant death up to day 6, and name of congenital disease.

The author used case reports on birth defects or congenital anomaly data from 2005–2009 as the initial information for the present secondary data analyses. The mean response rate throughout the 5 years was 99.0% (3,026/3,056), meaning that almost a complete database reflecting the current situation of ART in Japan could be constructed.

All live births after ART were analyzed in the present study. The types of defects were reclassified according to the International Classification of Diseases, tenth edition (ICD-10, 2003 version). Diseases that were classified in the category of ICD-10 code Q00-Q99 (i.e., congenital malformations, deformations, and chromosomal abnormalities) were regarded as birth defects in the present study. CH was classified as part of E00 (congenital iodine-deficiency syndrome) or E03 (other hypothyroidism). The present data did not allow the author to distinguish between permanent CH and transient CH. Thus, all types of CH were treated as CH in the present study. The total number of ART live births, singletons and multiples, were available from 2007 to 2009. The author estimated the number of ART singletons and multiples between 2005 and 2006 using approximation formulae [29].

### Statistical analyses

All patients with CH were listed with their obstetric data and neonatal outcome to further examine the features of CH patients. The frequency of additional birth defects in neonates with CH was calculated. Next, the relative risk (RR: the incidence rate of multiples/singletons) with the corresponding 95% confidence interval (CI) was calculated with singletons as the reference group.

Finally, the familial aggregation of CH was analyzed. The probandwise concordance rate [30] was calculated. The probandwise concordance rate is in a restricted sense the probability that a twin is affected given that his/her co-twin is affected. These rates can be compared directly to risk rates reported for other familial pairings and to population incidence rate figures [30]. Probandwise concordance rates were calculated as 2×C/(2×C+D), assuming complete and double ascertainment, where C denotes the number of affected concordant pairs and D denotes the number of discordant pairs [30]. For the triplets, the neonates were counted as concordant only when all neonates have CH in the present study.

A widely used measure of familial aggregation is the sibling recurrence risk ratio (RRR), defined as the ratio of the risk of disease manifestation, given that one’s sibling is affected, compared with the disease incidence rate in the general population [31,32]. The probandwise concordance rate of multiple births was divided by the incidence rate of the CH in the total ART population or the Japanese general population in this study.

Statistical analysis was conducted using Microsoft Excel 2010 and SAS for Windows ver. 9.2.

## Results

Demographic and perinatal outcome data of all neonates with CH are presented in Table 1. There were 18 patients total, consisting of 12 singletons and 6 multiples. The percentages of preterm delivery in singletons and multiples were 9% (=1/11) and 100% (=5/5), respectively. The sex ratio was 1 (9 males and 9 females). Additional birth defects were present in three patients with CH (17%=3/18). Multiples were more frequently affected by other birth defects (33%=2/6) than singletons (8%=1/12). The authors of previous studies noted that patent ductus arteriosus (PDA) is related to prematurity and is consequently more prevalent in twins [33,34]. When the frequency was calculated by excluding PDA, the result was 11% (=2/18). Multiples were also more frequently affected by other birth defects (17%=1/6) than singletons (8%=1/12).

**Table 1:** Demographic and perinatal outcome data of all neonates with CH

**ID**	**Maternal age**	**Method of ART**	**Blastocyst transfer**	**Gestational weeks**	**Plurality**	**Concordance/ Discordance in multiples**	**Sex**	**Early neonatal death**	**Other birth defects**
1	24	FET	no	37	singleton		male	no	
2	39	IVF	no	25	triplet	discordance	female	no	
3	33	IVF	yes	41	singleton		male	unknown	
4	39	ICSI	no	unknown	singleton		male	unknown	
5	31	ICSI	yes	35	twin	discordance	male	no	hypospadias
6	31	FET	no	27	twin	concordance	male	no	
7	31	FET	no	27	twin	concordance	male	no	patent ductus arteriosus
8	30	IVF	no	39	singleton		male	unknown	
9	33	ICSI	yes	40	singleton		female	no	
10	37	FET	yes	40	singleton		female	no	
11	28	ICSI	no	39	singleton		female	no	cleft lip, congenital genu recurvatum
12	40	FET	yes	41	singleton		female	no	
13	36	IVF	no	36	twin	discordance	male	no	
14	29	ICSI	no	34	twin	discordance	female	no	
15	39	IVF, ICSI	yes	36	singleton		female	no	
16	38	ICSI	no	40	singleton		female	no	
17	41	FET	yes	37	singleton		male	no	
18	33	FET	yes	37	singleton		female	no	

CH: congenital hypothyroidism, ART: assisted reproductive technology, FET: frozen embryo transfer, IVF: in-vitro fertilization, ICSI: intracytoplasmic sperm injection.

The incidence rate of CH per 1,000 live births was 0.17 (=18/106,678). The rate of CH was more than twofold higher in multiple births (0.31 ‰=6/19,533) than singleton births (0.14 ‰=12/87,145), but the difference was not statistically significant (RR=2.2, 95% CI 0.8–5.9). The proportion of multiple births observed in the CH patients (33%=6/18) was twofold higher than that estimated in the total ART population (18%=19,533/106,678) in the same period (2005–2009).

The calculated concordance rate and RRR are presented in Table 2. Six multiple-births patients were derived from one concordant twin pair, which consisted of two twin patients; three discordant twin pairs, which consisted of three twin patients; and one discordant triplets set, which consisted of one triplet patient. Thus, the probandwise concordance rate was 33.3% (=(2×1)/(2×1+4)). The estimated RRR was 1976 for the total ART population or 609 for the general Japanese population.

**Table 2:** Concordance rate and RRR of CH, 2005-2009

**Multiples**			**Total ART population**		**RRR(=A**/**X)**	**Japanese general population**		**RRR (=A/Y)**
**Concordant**	**Discordant**	**Probandwise concordance rate (A)(%)**	**N**	**Incidence rate ( ‰) (X**)		**N**	**Incidence rate( ‰) (Y)**	
**pair(N)**	**pair or set(N)**							
1	4	33.3	18	0.17	1976	2956	0.55	609

RRR: recurrence risk ratio, CH: congenital hypothyroidism, ART: assisted reproductive technology.

RRR was calculated probandwise concordance rate divided by the incidence rate in the general population.

The incidence rate in the Japanese general population was calculated using the data presented by the Japan Child and Family Research Institute (http://www.boshiaiikukai.jp/img/milk/kensajokyoH22.pdf (In Japanese). Accessed December 23, 2012). The prevalence throughout 2005–2009 was 0.55 ‰ (=2,956/5,398,934).

Probandwise concordance rate was calculated as 33.3%=2/(2+4).

RRR for total ART population was calculated as 1976=(2/6)/(18/106,678).

RRR for Japanese general population was calculated as 609=(2/6)/(2,956/5,398,934).

## Discussion

### Incidence rate and RR

To my knowledge, there has been no published study in which the authors directly analyzed the effect of ART on CH. CH incidence rate in developed countries is usually estimated by the data from neonatal screening tests, which are performed after birth. The incidence rate of CH noted in the present study is lower than that noted in many other studies published recently. A neonatal screening test for CH has been mandatory in Japan since 1979. According to the Japanese data on CH, the mean incidence rate of CH was 1/3,600 between 1979 and 2004, and 1/2,000 in 2005, and it then increased slightly to 1/1,800 in 2009 (presented by the Japan Child and Family Research Institute, http://www.boshiaiikukai.jp/img/milk/kensajokyoH22.pdf (In Japanese). Accessed December 23, 2012). The lower incidence rate of CH in the present study might be attributed to the incomplete reporting. The follow-up period of the present data did not necessarily reflect the result of the mass screening test. Moreover, some obstetricians might not regard CH as a birth defect in the narrowest sense (namely, based on ICD-10). Nevertheless, the objective of this study was to evaluate the incidence rate of CH in multiple births compared to singletons, and not to compare the CH incidence rate across different populations. Therefore, the comparison of CH in multiple births and singletons maybe biased only if there is differential reporting according to plurality, which is not likely to have occurred.

In the present study, the incidence rate of CH in multiples was about twofold higher than singletons, and even though the difference was not statistically significant, the finding suggests that multiple birth is one of the risk factors of CH. According to the recent population-based case control study by Medda et al. [10], an increased risk for permanent CH was detected in twins by a multivariate analysis (RR=12.2, 95% CI 2.4–62.3). According to the Harris and Pass study in New York [6], the incidence of CH was nearly double in twin births (1:876) as compared to singletons (1:1765) and even higher with triplets/+ (1:575) in 2002–2003. According to the population-based study by Olivieri et al. [11], a more than 3-fold higher frequency of multiples was found in the CH population (10.1 in 10,000) than in the general population (3.2 in 10,000 live births). As is well known, multiple births occur far more often in ART than in cases of spontaneous conception in almost all developed countries [29]. The multiple-birth rate (per 1,000 live births) was increased nearly doubled (12.4 in 1986 and 22.7 in 2005) in Japan, mainly due to iatrogenic multiple births of advanced-age mothers [29]. Thus, the widespread use of fertility treatment, including ART, might in part have contributed to the rise in the total incidence rate of CH in the general population, by indirectly increasing multiple births. Unfortunately, the present results did not reflect this hypothesis.

### Additional birth defects

Many studies [8,12-23] reported that the frequencies of additional birth defects with CH were between 8% and 20%, except that of a recent study by Reddy (59%) [24]. The present result (17%) was well within this range. The present results also suggested that this tendency is more obvious in multiples than in singletons. The mechanism of a higher frequency of additional birth defects in patients with CH was unclear. Olivieri et al. [11] proposed the hypothesis that genes involved in the development of the thyroid and other organs may be affected during the early stages of embryogenesis. However, it should be noted with caution that permanent CH showed a lower frequency of additional birth defects than did transient CH [18].

It has been well established that congenital heart disease is the most frequently occurring additional birth defect in patients with CH [8,14,17,21-25]. In the present study, PDA was found in one twin. A recent study by Reddy et al. [24] reported that two out of ten patients had PDA in addition to CH. Other birth defects with CH in the present patients were hypospadias, cleft lip, and congenital genu recurvatum. Olivieri et al. [22] performed a population-based study of the frequency of additional birth defects in patients with CH. Cleft lip, urological malformation, and musculoskeletal anomalies were reported as additional birth defects in patients with CH, although the numbers were small [22]. The present results might be supported by these findings.

### Familial aggregation

There have been few genetic epidemiologic studies on CH. Family study is a useful tool to show familial aggregation. Intensive family studies on CH were performed by a French group [27,28,35] who found that approximately 2% of CH cases with thyroid dysgenesis were familial. Although familial cases represent a minority of cases of CH caused by thyroid disgenesis, such cases were observed in more than 15-fold higher proportion than would be expected from chance alone, suggesting genetic factors.

Apart from case reports, the only population-based twin study was performed by Olivieri et al. [11]. According to their study, the pairwise concordance rate of twin pairs with unknown zygosity for permanent CH was low (4.3%=3/70) and was due to there being three pairs. They suggested that the sporadic occurrence of CH was likely due to noninheritable postzygotic events that may have included epigenetic modifications and early somatic mutation. We view their results with caution because their calculation was based on a pairwise, not probandwise method. The probandwise concordance rate of their data was recalculated as 8.2% (=6/73). This value would be compared with the incidence rate in the general population, or would be calculated according to the zygosity of twin pairs. Using the probandwise concordance rate and their incidence rate in the general population (0.032% in singletons), the RRR of twins was 256, which might be high enough to suggest familial aggregation. Olivieri et al. [11] also found a high recurrence risk (35.4) for CH in siblings of affected babies and indicated that environmental risk factors may act as a trigger in persons with a susceptible genetic background. There was one concordant pair in the present study, which produced a relatively high probandwise concordance rate (33.3%) and RRR. Although the present concordance rates and RRR were influenced by chance factors, familial aggregation of CH was suggested.

Population-based twin study using univariate/multivariate genetic analyses based on the structural equation modeling is the most powerful tool to clarify the genetic/environmental contribution to CH and comorbidity with other birth defects [36]. Record linkage between a mass screening registry of CH and twin registry would make this possible.

### Limitations

This study has the following limitations, most of which could be attributed to the dataset, based on the fact that individual information was obtained only from the subjects with birth defects and/or CH after ART, not the total ART live births. The first limitation is that the author could not check the reliability of the data directly. This is the essential limitation of secondary data analyses. Second, although the present dataset was from a multi-year nationwide survey, it still did not have sufficiently high statistical power to detect statistical significance. Third, the author could not control for confounding factors that can affect ART and/or CH. Fourth, follow-up after birth was limited to the neonatal period at the latest, and was incomplete [29]. Both CH and some birth defects are not obvious within a few days after birth. Fifth, the CH could not be distinguished between permanent and transient type. From the view of disease prevention, all types of CH should be properly followed up. This problem should also be discussed from the viewpoint of medical economics for mass screening of CH.

## Conclusions

CH was more frequent in multiples compared to singletons. A higher percentage of additional birth defects was also observed in multiples compared to singletons. The familial aggregation of CH was suggested.

## Abbreviations

CH: Congenital hypothyroidism; ART: Assisted reproductive technology; RR: Relative risk; CI: Confidence interval; RRR: Recurrence risk ratio; PDA: Patent ductus arteriosus; FET: Frozen embryo transfer; IVF: In-vitro fertilization; ICSI: Intracytoplasmic sperm injection.

## Competing interests

The author declare that I have no competing interests.

## Author’ contributions

SO carried out data gathering, analyses, and writing of manuscript.
